# The association of urinary epidermal growth factors with ADPKD disease severity and progression

**DOI:** 10.1093/ndt/gfad050

**Published:** 2023-03-13

**Authors:** Laura R Harskamp, Maria Vanessa Perez-Gomez, Judith E Heida, Gerwin E Engels, Harry van Goor, Marius C van den Heuvel, Andrew J Streets, Albert C M Ong, Alberto Ortiz, Ron T Gansevoort, the DIPAK Consortium, J P H Drenth, J W de Fijter, M D A van Gastel, E Meijer, M Losekoot, D J M Peters, F W Visser, J Wetzels, R Zietse

**Affiliations:** Department of Nephrology, University Medical Center of Groningen, University of Groningen, Groningen, The Netherlands; Department of Nephrology, Fundación Jiménez Díaz University Hospital and IIS-FJD, Madrid, Spain; Department of Nephrology, University Medical Center of Groningen, University of Groningen, Groningen, The Netherlands; Haemoscan BV, Groningen, The Netherlands; Department of Pathology and Medical Biology, University Medical Center of Groningen, University of Groningen, Groningen, The Netherlands; Department of Pathology and Medical Biology, University Medical Center of Groningen, University of Groningen, Groningen, The Netherlands; Department of Infection, Immunity and Cardiovascular Disease, University of Sheffield Medical School, Kidney Genetics Group, Academic Nephrology Unit, Sheffield, UK; Department of Infection, Immunity and Cardiovascular Disease, University of Sheffield Medical School, Kidney Genetics Group, Academic Nephrology Unit, Sheffield, UK; Department of Nephrology, Fundación Jiménez Díaz University Hospital and IIS-FJD, Madrid, Spain; Department of Nephrology, University Medical Center of Groningen, University of Groningen, Groningen, The Netherlands

**Keywords:** ADPKD, EGF, EGFR, eGFR, repair

## Abstract

**Background:**

The epidermal growth factor receptor (EGFR) pathway is involved in kidney tissue repair and growth. Preclinical interventional data and scarce human data have suggested a role for this pathway in the pathophysiology of autosomal dominant polycystic kidney disease (ADPKD), while other data have suggested that its activation is causally linked to repair of damaged kidney tissue. We hypothesize that urinary EGFR ligands, as a reflection of EGFR activity, are associated with kidney function decline in ADPKD in the context of tissue repair following injury, and as the disease progresses as a sign of insufficient repair.

**Methods:**

In the present study, we measured the EGFR ligands, EGF and heparin binding-EGF (HB-EGF), in 24-h urine samples of 301 ADPKD patients and 72 age- and sex-matched living kidney donors to dissect the role of the EGFR pathway in ADPKD. During a median follow-up of 2.5 years, the association of urinary EGFR ligand excretion with annual change in estimated glomerular filtration rate (eGFR) and height-adjusted total kidney volume in ADPKD patients was analyzed using mixed-models methods, and the expression of three closely related EGFR family receptors in ADPKD kidney tissue was investigated by immunohistochemistry. Additionally, the effect of reducing renal mass (after kidney donation), was assessed to investigate whether urinary EGF matches this reduction and thus reflects the amount of remaining healthy kidney tissue.

**Results:**

At baseline, urinary HB-EGF did not differ between ADPKD patients and healthy controls (*P* = .6), whereas a lower urinary EGF excretion was observed in ADPKD patients [18.6 (11.8–27.8)] compared with healthy controls [51.0 (34.9–65.4) μg/24 h, *P* < .001]. Urinary EGF was positively associated with baseline eGFR (R = 0.54, *P* < .001) and a lower EGF was strongly associated with a more rapid GFR decline, even when adjusted for ADPKD severity markers (β = 1.96, *P* < .001), whereas HB-EGF was not. Expression of the EGFR, but not other EGFR-related receptors, was observed in renal cysts but was absent in non-ADPKD kidney tissue. Finally, unilateral nephrectomy resulted in a decrease of 46.4 (–63.3 to –17.6) % in urinary EGF excretion, alongside a decrease of 35.2 ± 7.2% in eGFR and 36.8 ± 6.9% in measured GFR (mGFR), whereas maximal mGFR (measured after dopamine induced hyperperfusion) decreased by 46.1 ± 7.8% (all *P* < .001).

**Conclusions:**

Our data suggest that lower urinary EGF excretion may be a valuable novel predictor for kidney function decline in patients with ADPKD.

KEY LEARNING POINTS **What is already known about this subject?**

- Preclinical interventional data and scarce human data have suggested a role for the epidermal growth factor receptor (EGFR) pathway in the pathophysiology of autosomal dominant polycystic kidney disease (ADPKD).

**What this study adds?**

- A small cross-sectional study showed that lower urinary EGF excretion is associated with more severe disease, but its suitability as biomarker predicting ADPKD outcome has not been investigated yet.
- Our study shows that lower urinary EGF excretion is strongly and independently associated with more severe disease at baseline, as well as with more rapid eGFR loss during follow-up in patients with ADPKD.

**What impact this may have on practice or policy?**

- These data indicate that lower urinary EGF excretion may be a valuable biomarker to predict future kidney function decline in patients with ADPKD.

## INTRODUCTION

In patients with autosomal dominant polycystic kidney disease (ADPKD) [1] innumerable renal cysts develop during aging that grow and gradually replace healthy kidney tissue, leading to an impaired kidney function. The molecular pathways underlying cyst formation have been of great interest to researchers over the years, because gaining a better understanding of them may result in development of novel therapeutic interventions and biomarkers for prediction of disease progression.

A pathway of particular interest is the one mediated by the epidermal growth factor receptor (EGFR) family. When these receptors are activated by theirs ligands, a variety of intracellular signals are triggered, which in normal conditions regulate growth, migration and repair of kidney cells [2]. Since EGFR activation leads to cell proliferation, it has been suggested that the EGFR pathway may play a role in disease progression in ADPKD [3]. Several studies have been published that support this hypothesis. Expression of the EGFR is increased in polycystic kidney disease (PKD) renal tissue [4]. *In vitro* and *in vivo* experiments have demonstrated that activation of the EGFR pathway leads to cyst development [5, 6], whereas pharmacological targeting of the EGFR pathway resulted in reduced kidney growth in animals models [7, 8]. Furthermore, a human cross-sectional study showed that urinary EGFR ligands were associated with disease severity in ADPKD [9]. Whether activity of the EGFR pathway is associated with progression of ADPKD in a longitudinal setting has not been studied.

Despite ample evidence linking EGFR activation to aberrant kidney growth in ADPKD, other data have suggested that this pathway has an important beneficial influence on kidney health, given that it is involved in regeneration and repair of damaged kidney tissue [10, 11]. This ambiguity in the role of the EGFR pathway, especially in ADPKD, has yet not been fully resolved.

Given these considerations, we investigated in the present study whether EGFR pathway activation is associated with disease progression during follow-up in a cohort of ADPKD patients, and whether such an association reflects a role in cyst growth or repair. EGFR pathway activation was measured by analyzing urinary excretion of two ligands of the EGFR family, EGF and heparin-binding EGF-like growth factor (HB-EGF), and by expression of three receptors of the EGFR family, the EGFR (ErbB1/HER1), ErbB2 (HER2/neu) and ErbB4 (HER4).

## MATERIALS AND METHODS

### Study population

We included patients who participated in the Developing Interventions Strategies to Halt Progression of Autosomal Dominant Polycystic Kidney Disease (DIPAK)-1 trial, an investigator-driven, open-label clinical trial including 309 ADPKD patients who were randomized to receive the somatostatin analogue lanreotide on top of standard care or standard of care alone. Primary outcome was change in slope of estimated glomerular filtration rate (eGFR) during a 2.5-year treatment period. Design, methods and main outcomes of the DIPAK-1 trial have been published elsewhere [12, 13]. In brief, patients were included between 2012 and 2015 if they were 18 to 60 years of age, had ADPKD diagnosed by the modified Ravine criteria [14] and an eGFR between 30 to 60 mL/min/1.73 m^2^. After a baseline visit, participants were followed for 132 weeks during which patients were seen every 12 weeks. Exclusion criteria were bradycardia, a history of gallstones or pancreatitis and concomitant diseases or use of medication that may influence the natural course of ADPKD (e.g. diabetes mellitus, use of tolvaptan or non-steroidal anti-inflammatory drugs). The DIPAK-1 trial was a multicenter effort by the University Medical Centers of Groningen, Leiden, Nijmegen and Rotterdam. The present study included 301 out of the 309 participants who had frozen baseline urine samples available for analysis.

Baseline results of the ADPKD patients were compared with a healthy control group consisting of 72 age- and sex-matched (4:1) living kidney donors selected from an observational clinical cohort that included all individuals who were accepted in the living kidney donation program of the University Medical Center Groningen between 1996 and 2007 (*N* = 253). None had a history of kidney disease, diabetes or cardiovascular events, and all were normotensive. Details of this cohort have been published previously [15].

Both studies were approved by the ethical board of the University Medical Center Groningen (METc 2012/060; METc 2014/077, respectively) and were conducted in agreement with the Declaration of Helsinki. All participants gave written informed consent.

Kidney samples were obtained from 19 patients with ADPKD of the DIPAK Biobank for human ADPKD tissue, which collected samples from 2013 until 2015 from patients who underwent a scheduled unilateral or bilateral nephrectomy, mostly prior to kidney transplantation, in whom space must be made to implant the graft, while the remaining patients underwent nephrectomy because of complications such as recurrent cyst hemorrhage, cyst infections and mechanical issues due to the kidney enlargement. Inclusion criteria were patients diagnosed with ADPKD based upon the Ravine criteria and aged between 18 and 80 years. The only exclusion criterion was the start of kidney function replacement therapy prior to surgery. All patients had impaired kidney function and most had reached the KDIGO chronic kidney disease stage G5 (kidney failure).

Control specimens were taken from healthy parts of kidneys from 12 patients who underwent surgery for renal tumors and who had a normal kidney function (eGFR >60 mL/min/1.73 m^2^). Moreover, kidney tissue from five subjects with an impaired kidney function were investigated (with hydronephrosis and reflux nephropathy).

All procedures and use of anonymized tissue were performed according to national ethical guidelines.

### Biochemical measurements and clinical assessments

A detailed description of both study protocols has been published previously [9]. All subjects included in the DIPAK study completed a 24-h urine collection the day before their baseline visit, and also after 12 weeks. Living donors collected 24-h urine samples before and after donation. All samples were stored frozen at –80°C for several years until measurement. As shown previously (PMID 30835251), urinary EGF and HB-EGF remain stable under these storage conditions. Urinary EGF and HB-EGF concentrations were measured in these samples with optimized duo set enzyme-linked immunosorbent assays (R&D Systems, Minneapolis, MN, USA) by Heamoscan BV, The Netherlands, as described previously [9]. The lower limit of detection with the modified assays for EGF is 1.7 ng/mL, and for HB-EGF 6.3 pg/mL. Median interassay coefficients of variation of urinary EGF and HB-EGF in ADPKD patients were 10.5% and 14.0%, respectively. Urinary ligand excretions were calculated as urinary ligand concentration × 24-h urine volume.

Medical history, biometric data and blood samples of the ADPKD patients were obtained during their respective study visits. GFR was estimated with the Chronic Kidney Disease Epidemiology Collaboration (CKD-EPI) equation [16] with creatinine measured with an enzymatic assay (isotopedilution mass spectrometry traceable; Modular, Roche Diagnostics). T2-weighted coronal magnetic resonance images obtained without contrast agent were used to measure total kidney volume (TKV) at baseline and at the end-of-treatment visit by manual tracing of the kidney outline by trained personnel (AnalyzeDirect, Inc., Overland Park, KS, USA) [17]. To correct for gender, TKV was adjusted for height (htTKV). Thereafter, the Mayo htTKV risk class was ascertained [18]. *PKD* mutations were screened in genomic DNA using locus-specific long-range amplification followed by direct Sanger sequencing of exonic and flanking intronic regions of *PKD1* and *PKD2, HNF1B, PKHD1*, and *GANAB*, combined with MLPA for larger deletions/duplications [13, 19]. Alternatively, a gene panel–based NGS approach was used analyzing 137 genes or a capture with the Agilent SureSelectXT Inherited Disease Panel kit and analysis of 78 genes [20]. As urinary kidney damage biomarkers, we measured albumin, immunoglobulin G, β2-microglobulin (β2MG), kidney injury molecule-1 (KIM-1), heart-type fatty acid binding protein (HFABP), neutrophil gelatinase-associated lipocalin (NGAL) and monocyte chemotactic protein 1 (MCP-1) in 24-h urine samples. Details of these assays have been previously described [21].

For the healthy kidney donors, height, weight, blood pressure and measured glomerular filtration rate (mGFR) were measured as the urinary clearance of ^125^I-iothalamate administered by continuous infusion at approximately 3 months before and at 3 months after living kidney donation as part of the routine screening and post donation evaluation program. Renal functional reserve (RFR_dopa_) was measured by extending the procedure mentioned above with 2 h of a 1.5 μg/kg/min of dopamine infusion, and defined as mGFR_dopamine_ − mGFR.

### Tissue preparation and immunohistochemistry

All kidney tissue was fixed in 4% paraformaldehyde and processed for paraffin wax embedding according to standard procedures. Routine morphology was evaluated using hematoxylin and eosin-stained sections by an experienced renal pathologist. Tissue of the patients with ADPKD was selected based upon the presence of glomeruli, tubules and multiple cysts in different stages of development. Control specimens were only used if characterized macroscopically as non-diseased.

Using immunohistochemistry, consecutive sections were stained for the various EGFR family receptors. Therefore, tissues were de-waxed and subjected to heat‐induced antigen retrieval by incubation in 10 mM citrate (pH 6) buffer at a power of 500 Watt for 15 min in the microwave. Endogenous peroxidase was blocked with 500 μL 30% H_2_O_2_ in 50 mL phosphate‐buffered saline (PBS, pH 7.4) for 30 min. Antibody dilutions were made in PBS supplemented with 1% bovine serum albumin.

A monoclonal mouse anti-phosphorylated EGFR antibody (Tyr-1068, Invitrogen) diluted in PBS and supplemented with 1% bovine serum albumin was used at a concentration of 1:200 for 1 h at room temperature. For ErbB2 and ErbB4, a monoclonal rabbit antibody anti-ErbB2 (Invitrogen) at a concentration of 1:40, and a monoclonal rabbit anti-phosphorylated ErbB4 antibody (phosphor Y1284, Abcam) at a concentration of 1:100 were used, respectively. Antibody binding was detected using sequential incubations with peroxidase-labeled mouse anti-rabbit and peroxidase-labeled rabbit anti-goat (RAMPO/GARPO; Dako, Glostrup, Denmark) for EGFR, and with peroxidase-labeled rabbit anti-goat and goat anti-rabbit for both ErbB2 and ErbB4 (GARPO/RAGPO, Dako, Glostrup, Denmark), respectively. Human antibody serum (1%) was added to the secondary antibodies. Peroxidase activity was developed by using 3,3′-diaminobenzidine tetrachloride (DAB) for 10 min. Counterstaining was performed using Mayer's hematoxylin. A control procedure was performed to determine the specificity of the antibody. Sections incubated with PBS without the primary antibodies were consistently negative. The renal pathologist analyzed the stained sections for structures positive for phosphorylated EGFR, ErbB2 and ErbB4 receptor.

### Statistical analyses

Data are reported as mean (standard deviation) for normally distributed variables and median (interquartile range) for skewed data. Binary variables are shown as number (%). Differences in baseline characteristics, urinary EGF excretion and urinary HB-EGF excretion between the patients with ADPKD and healthy controls were tested using Students’ *t*-tests for parametric data, Mann–Whitney U tests for non-parametric data and chi-square tests for categorical data. We used univariable and multivariable linear regression analysis for cross-sectional associations of baseline urinary EGF and HB-EGF excretion with ADPKD severity markers, i.e. eGFR, htTKV and urinary tubular damage markers.

For longitudinal analyses, we only studied patients who were randomized to standard of care as lanreotide may influence disease progression. A mixed-model repeated measures analysis was used to calculate annual change in eGFR per patient. Associations between urinary EGF and HB-EGF excretions and slope of kidney function decline were adjusted for age, sex, body mass index, baseline eGFR, htTKV, *PKD* mutation and the urinary excretion of the tubular damage markers β2MG, HFAPB and MCP-1 (the most important damage markers in ADPKD in a previous study). In addition, we tested interaction terms between these adjustment factors and the urinary EGFR ligand excretions as determinants of rate of kidney function decline.

Various sensitivity analyses were performed. First, we repeated the above-mentioned analyses incorporating also participants treated with lanreotide. Second, disease progression was also studied as slope of htTKV, by calculating the annual growth in kidney volume from the difference between baseline and end-of-study visit with the following formula: [(TKV last study visit/TKV baseline visit)^1/total study time^] – 1*100. Third, in order to eliminate an effect of incomplete 24-h urine collections, the above-mentioned analysis was repeated for urinary excretions of EGF and HB-EGF corrected for creatinine excretion. Fourth, the urinary excretion of the EGFR ligands at baseline and at 12 weeks were compared in the standard of care subgroup of the DIPAK-1 trial to ascertain the consistency of urinary EGF and HB-EGF measurement over time. Finally, urinary EGF and HB-EGF excretion before and after kidney donation were compared by means of Wilcoxon signed rank tests for paired analysis to evaluate the effect of a change of healthy kidney mass on the excretion of these ligands.

Skewed variables were natural log transformed for the aforementioned analyses. Analyses were performed with SPSS version 23.0 (SPSS Inc., Chicago, IL, USA) and STATA version 14 (Stat Corp., College Station, TX, USA). A two-sided *P* < .05 was considered statistically significant. Digital images of the renal tissues were obtained with Eperio or Hammamatsu scanners, and viewed with ImageScope version 12.01.

## RESULTS

### Baseline measurements

Baseline characteristics of the 301 patients with ADPKD and 72 age- and sex-matched healthy controls are presented in Table 1. There were no significant differences between these two groups of subjects except that patients had a higher systolic and diastolic blood pressure (133 over 82 versus 126 over 77 mmHg, respectively, both *P* < .001), were more likely to use blood pressure–lowering medication (91 versus 13%, *P* < .001) and had a lower baseline eGFR (51.7 versus 94.5 mL/min/1.73m^2^, *P* < .001), reflecting their disease status.

**Table 1:** Baseline characteristics of ADPKD patients and healthy controls.

	ADPKD patients	Healthy controls	*P*-value
Number	301	72	
Age (years)	48 ± 7.3	51 ± 9.8	.09
Sex (% male)	45.8	50.0	.60
BMI (kg/m^2^)	26.0 (23.9–29.3)	25.5 (23.7–28.3)	.32
SBP (mmHg)	133 ± 13.3	126 ± 15.3	<.001
DBP (mmHg)	82 ± 9.5	77 ± 9.5	<.001
Use of antihypertensive drugs (% yes)	91	13	<.001
PKD genotype [*n* (%)]			
PKD1 truncating	144 (49)		
PKD1 non-truncating	73 (25)		
PKD2	60 (21)		
Others	15 (5)		
htTKV (mL/m)	1084 (754–1658)		
Mayo htTKV class [*n* (%)]			
1A	5 (2)		
1B	44 (15)		
1C	108 (37)		
1D	78 (26)		
1E	50 (17)		
2	10 (3)		
eGFR (mL/min/1.73 m^2^)	51.7 ± 11.6	94.5 ± 13.1	<.001
mGFR (mL/min/1.73 m^2^)		104.9 ± 16.7	
Urinary EGF excretion (μg/24 h)	18.6 (11.8–27.8)	51.0 (34.9–65.4)	<.001
Urinary EGF/creatinine ratio (μg/mmol)	1.4 (1.0–2.2)	3.8 (2.8–5.6)	<.001
Urinary HB-EGF excretion (ng/24 h)	176 ± 101	170 ± 88	.64
Urinary HB-EGF/creatinine ratio (ng/mmol)	14.1 ± 8.9	13.9 ± 8.6	.87

Variables are presented as mean ± SD when normally distributed and as median (interquartile range) when not-normally distributed.

eGFR calculated using the CKD-EPI equation; mGFR measured using ^125^I-iothalamate.

BMI, body mass index; SBP, systolic blood pressure; DBP, diastolic blood pressure; uHB-EGF, urinary heparin-binding EGF-like growth factor; uEGF, urinary epidermal growth factor.

Table 1 shows that in patients with ADPKD, urinary EGF excretion was lower [18.6 (11.8–27.8) μg/24 h] compared with healthy controls [51.0 (34.9–65.4) μg/24 h, *P* < .001], whereas urinary HB-EGF excretion was similar between the two study groups (176 ± 101 versus 170 ± 88 ng/24 h, *P* = 0.64). Correcting urinary excretion of these markers for creatinine excretion rendered essentially similar results [EGF 1.4 (1.0–2.2) versus 3.8 (2.8–5.6) μg/mmol, respectively, *P* < .001, and HB-EGF 14.1 ± 8.9 versus 13.9 ± 8.6 ng/mmol, respectively, *P* = 0.87].

The renal histology subset of patients selected from the DIPAK Biobank for renal tissue had a mean age of 49.8 ± 9.8 years, eGFR of 9.5 (7.8–14.8) mL/min/1.73 m^2^, and total kidney weight of 2704 (1584–5414) g due to their advanced stage of disease. Controls with preserved kidney function were aged 60 ± 11 years, in whom a kidney was removed because of a renal cancer (healthy tissue was used for our analyses), while controls with impaired kidney function (mean age 40 ± 29 years) had a kidney removed due to recurrent urinary tract infections (although not at the time of nephrectomy).

### Intra-individual stability over time of urinary EGF

**Figure 1:**
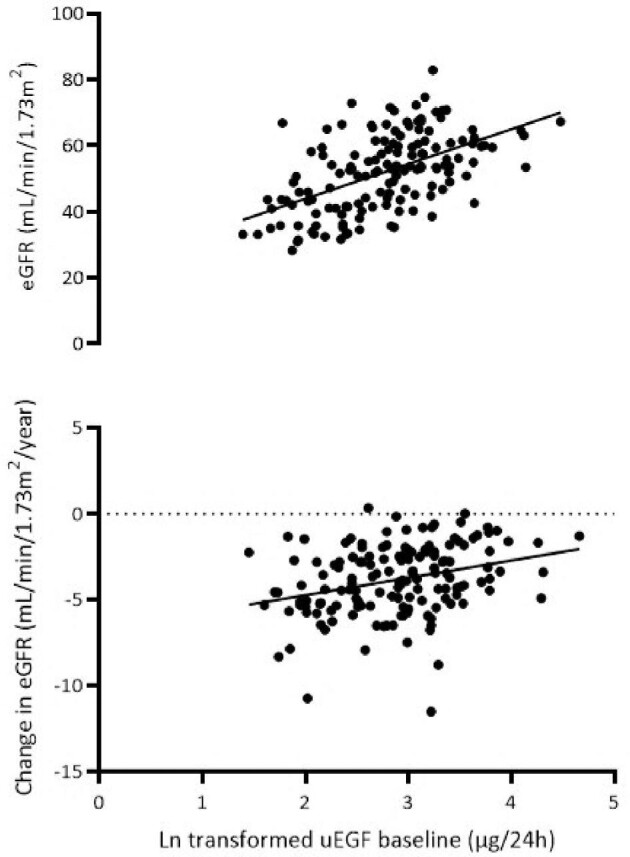
Association between urinary EGF and disease severity and progression.

To assess intra-individual stability over time, we measured urinary EGF excretion in patients randomized to standard of care at baseline and at Week 12 of the study (*n* = 149). Urinary EGF excretion was 18.8 (11.6–26.6) at baseline and 17.0 (11.4–25.2) μg/24 h at week 12 (*P* = .07). Figure 1 shows a strong correlation between EGF measurements at baseline and Week 12, mimicking the line of identity (R = 0.82 and *P* < .001). These data demonstrate that EGF can be measured accurately in urine.

### Urinary EGF and HB-EGF versus ADPKD severity

At baseline, we investigated associations between urinary EGF excretion and markers of disease severity in ADPKD patients (Table 2). Urinary EGF excretion was positively associated with eGFR (R = 0.54, *P* < .001). After adjusting for age, sex, htTKV, *PKD* mutation, and the urinary biomarkers β2MG, HFABP and MCP-1, the association of EGF with eGFR remained significant (R = 0.49, *P* < .001). For HB-EGF, opposite findings were seen (Supplementary data, Table S3A). With more severe disease (i.e. a lower eGFR), urinary HB-EGF was higher (R = –0.16, *P* = .007). Likewise, HB-EGF remained significantly associated with baseline eGFR after adjusting for age, sex, htTKV and *PKD* mutation (R = –0.14, *P* = .02). However, this association disappeared in the final model when urinary damage biomarkers were added (R = –0.001, *P* = .99).

**Table 2:** Association of uEGF with eGFR at baseline in 301 ADPKD patients.

	Model 1			Model 2			Model 3			Model 4		
	St. β	*P*-value	R^2^	St. β	*P*-value	R^2^	St. β	*P*-value	R^2^	St. β	*P*-value	R^2^
**eGFR (mL/min/1.73 m^2^)**			0.32			0.35			0.36			0.41
Ln uEGF (μg/24 h)	0.54	<.001		0.53	<.001		0.54	<.001		0.49	<.001	
Age (years)	–0.06	.23		–0.09	.07		–0.08	.14		–0.14	.02	
Female sex	0.10	.03		0.06	.25		0.05	.35		0.05	.33	
Ln htTKV (mL/m)				–0.17	.001		–0.16	.002		–0.08	.13	
*PKD* mutation												
*PKD1* truncating							–0.05	.48		–0.04	.53	
*PKD1* non-truncating							0.03	.60		0.03	.66	
Urinary damage markers												
β2MG (μg/24 h)										–0.10	.05	
HFABP (μg/24 h)^a^										–0.15	.003	
MCP-1 (ng/24 h)										–0.13	.04	

Linear regression analysis with dependent variables indicated in bold. Data were logarithmically (Ln) transformed if appropriate. Reference group for *PKD* mutation is *PKD*2 and others (non-*PKD* mutations) combined.

^a^One extreme outlier removed.

uEGF, urinary excretion of epidermal growth factor.

Neither urinary EGF nor HB-EGF were associated with htTKV in this ADPKD patient cohort, either uncorrected or after correction for additional predictors (Supplementary data, Table S2 and S3B). Of note, at baseline eGFR and TKV were significantly correlated (R = –0.18, *P* = .002).

### Urinary EGF and HB-EGF as predictors for ADPKD progression

**Figure 2:**
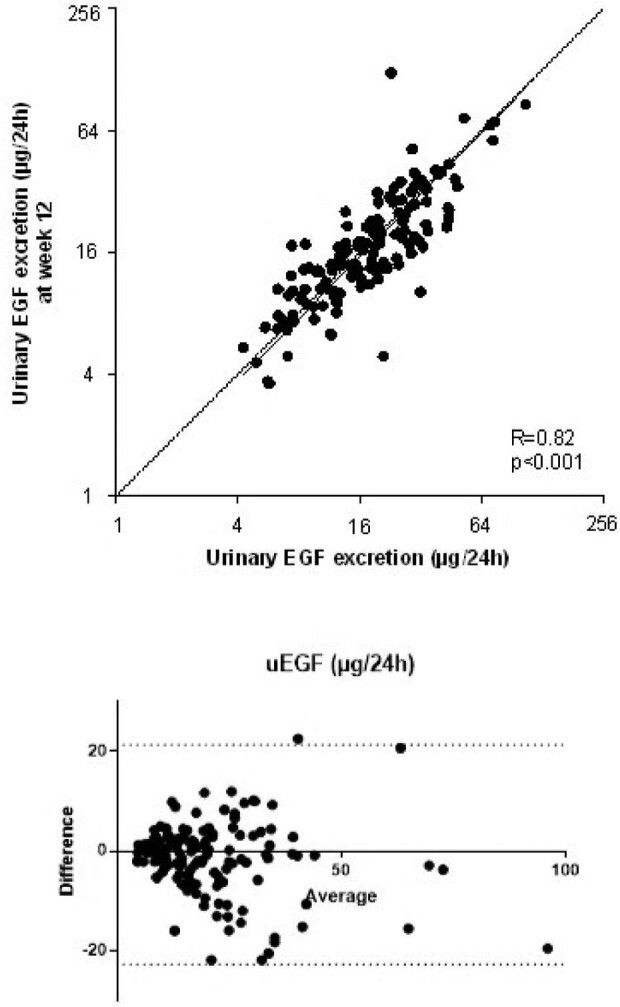
Consistency between urinary EGF excretion at baseline and at 3 months of follow-up. uEGF, urinary epidermal growth factor excretion. The upper panel shows a scatterplot in which the solid line is drawn with use of Deming regression and the dotted line represents the line of identity. The correlation coefficient was determined with Pearson's correlation statistics. The lower panel shows a Bland–Altman plot. Repeated measurement of uEGF showed a bias of –0.75 ± 11.3 (95% limits of agreement of –22.9 to 21.4).

Possible associations between urinary concentrations of the EGFR ligands with kidney function decline and TKV growth were assessed in patients with ADPKD randomized to the standard of care (*n* = 149). The baseline characteristics of this study arm can be found in Supplementary data, Table S4. During a mean follow-up of 2.4 ± 0.36 years, on average 15 eGFR values were obtained per patient, rendering an annual rate of eGFR decline of –3.85 ± 2.05 mL/min/1.73 m^2^ per year. Table 3 and Figure 2 shows that a higher baseline urinary EGF excretion is significantly associated with less kidney function decline (β = 1.08, *P* < .001). In addition, we assessed whether patient characteristics such as age, sex, body mass index, blood pressure, eGFR, htTKV, urine volume and urinary damage biomarkers significantly influenced this association. This was not the case (β = 1.96, *P* < .001, Table 3). The use of renin–angiotensin–aldosterone system inhibitors did not have an impact on the level of urinary EGF excretion (*P* = .32) nor did it have an interaction with urinary EGF excretion for the outcome eGFR decline (*P* = .41). The results were similar in subjects treated with lanreotide (Supplementary data, Table S5) or after correction for creatinine excretion (Supplementary data, Table S6). In contrast, urinary EGF was not associated with percentage change in TKV per year during the study (Supplementary data, Table S7).

**Table 3:** Association of baseline uEGF with rate of kidney function decline during follow-up in 149 ADPKD patients receiving standard of care.

	Crude		Model 1		Model 2		Model 3		Model 4		Model 5	
	β	*P*-value	β	*P*-value	β	*P*-value	β	*P*-value	β	*P*-value	β	*P*-value
**Change in eGFR (mL/min/1.73 m^2^/year**)												
Ln uEGF (μg/24 h)	1.08	.001	1.79	<.001	1.96	<.001	1.97	<.001	1.95	<.001	1.96	<.001
Age (per 10 years)			1.49	<.001	1.45	<.001	1.14	<.001	0.84	.01	0.59	.08
Female sex			0.12	.75	0.08	.83	–0.06	.88	0.09	.81	–0.03	.94
eGFR (per 10 mL/min/1.73 m^2^)					–0.15	.43	–0.22	.24	–0.16	.41	–0.36	.06
Mayo htTKV class												
1B + 1C							–1.48	.07	–1.56	.05	–1.04	.19
1D + 1E							–1.44	.008	–2.52	.004	–1.81	.04
*PKD* mutation												
*PKD1* truncating									–1.05	.04	–1.25	.01
*PKD1* non-truncating									–1.21	.03	–1.40	.01
Urinary damage markers												
β2MG (mcg/24 h)											–0.22	.24
HFABP (μg/24 h)											–0.64	.05
MCP-1 (ng/24 h)											–0.50	.04

Associations tested with mixed model analysis. uEGF was ln transformed to attain normal distribution. Reference groups are PKD2 and other (non-PKD1 mutations) combined, and Mayo htTKV class 2 and 1A combined.

uEGF, urinary EGF excretion.

Performing the same analyses for urinary HB-EGF excretion showed no association with parameters of ADPKD progression (i.e. eGFR decline or TKV growth) (Supplementary data, Tables S8 and S9).

### EGFR family receptor expression in renal tissue of controls and ADPKD patients

**Figure 3:**
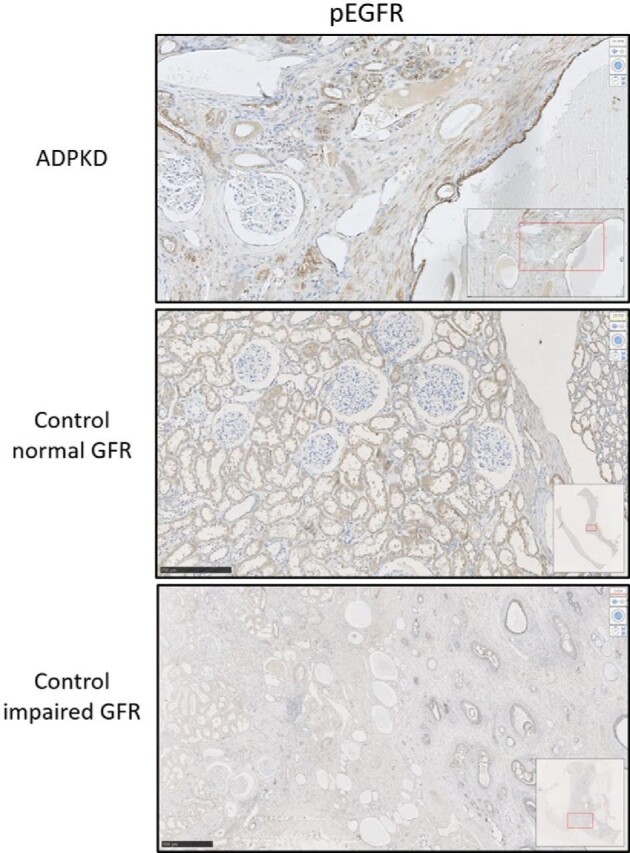
Representative IHC staining of EGFR expression in renal tissue of patients with ADPKD compared with controls with a preserved and impaired kidney function. IHC, immunohistochemistry.

Kidneys of healthy controls were all normal on routine morphological examination, whereas the kidneys of the controls with impaired kidney function showed inflammation and fibrosis due to obstruction. In both control groups, no glomerular or tubular expression of the phosphorylated EGFR, ErbB2 and ErbB4 was observed, except for some staining of phosphorylated EGFR in the collecting duct and phosphorylated ErbB4 in the distal tubules of controls with impaired kidney function (Table 4, Fig. 3). Morphological examination showed that the tissue slices of ADPKD patients consisted for large parts of fibrosis and cysts of varying sizes (1–10 mm), with limited normal tissue and mostly atrophic glomeruli due to the advanced disease stage of ADPKD in the patients for whom we had tissue. In the ADPKD slices, phosphorylated EGFR was present at cyst lining epithelial cells of most cysts at the apical cell membrane (Table 4), whereas phosphorylated ErbB2 was absent and phosphorylated ErbB4 was only present in some cysts (not shown). In addition, phosphorylated EGFR was expressed in the distal tubules and collecting duct of renal tissue of patients with ADPKD (Fig. 3), but not in glomeruli, whereas expression of phosphorylated ErbB2 and phosphorylated ErbB4 was not detected along the nephron (Table 4).

**Table 4:** IHC analyses of phosphorylated EGFR, ErbB2 and ErbB4 expression in renal tissue of patients with ADPKD (*n* = 19) compared with controls with a preserved (*n* = 12) and impaired kidney function (*n* = 5).

		ADPKD	Controls, normal eGFR	Controls, impaired eGFR
EGFR	Cysts	+		
	Glomerulus	–	–	–
	Proximal convoluted tubules	–	–	–
	Distal convoluted tubules	+	–	+/–
	Collecting duct	+	–	–
ErbB2	Cysts	–		
	Glomerulus	–	–	–
	Proximal convoluted tubules	–	–	–
	Distal convoluted tubules	+/–	–	–
	Collecting duct	–	–	–
ErbB4	Cysts	+/–		
	Glomerulus	–	–	–
	Proximal convoluted tubules	–	–	+/–
	Distal convoluted tubules	+/–	–	–
	Collecting duct	–	–	–

IHC staining was evaluated as the fraction of positive cells. If <50% of the fraction of the cells were stained positive they are depicted as missing (–) and if >50% of the fraction of cells were stained positive as present (+).

IHC, immunohistochemistry.

### Effect of uninephrectomy on urinary EGF excretion

The potential effect of uninephrectomy on urinary EGF excretion was studied in 72 healthy kidney donors controls by comparing these measurements before and after kidney donation. On average, there were 5.5 (4.0–7.0) months between these visits. After unilateral nephrectomy, eGFR and mGFR dropped by 35.2 ± 7.2% and 36.8 ± 6.9%, respectively, whereas maximal stimulated mGFR decreased by 46.1 ± 7.8% (all *P* < .0001). As shown in Supplementary data, Fig. S1, removing one kidney resulted in a decrease in urinary EGF from 51.0 (34.9–65.4) μg/24 h pre-donation to 26.3 (17.8–35.8) μg/24 h post-donation, a change of –46.4 (–63.3 to –17.6) % (*P* < .0001). Before and after kidney donation, urinary EGF correlated with maximal stimulated GFR (R = 0.41, *P* < .001, and R = 0.56, *P* < .001, respectively).

## DISCUSSION

In this study, we found a significantly lower 24-h urinary EGF excretion in patients with ADPKD compared with healthy controls, and a positive association between baseline urinary EGF and disease severity. These findings are consistent with our previous results in a smaller study cohort of 27 patients with ADPKD [7]. In addition, this study has shown that a lower urinary EGF excretion was associated with a more rapid decline in kidney function during a follow-up period of 2.5 years.

In contrast, urinary HB-EGF excretion was not different between patients and controls, and not associated with kidney function (decline). This difference between HB-EGF and EGF might be due to its production site; urinary EGF is solely produced in the kidney whereas HB-EGF is also produced elsewhere. HB-EGF may not be that important in the kidney, and specifically in ADPKD as thought. Our data suggest that only urinary EGF may serve as a potential marker to predict ADPKD disease progression.

Recent literature reveals that the effects of EGFR pathway activation on the kidney are complex. Some experimental studies have reported deleterious effects of EGFR activation in ADPKD, since EGFR blockade improved experimental progressive (polycystic) kidney disease [7, 8, 22 and 23]. On the other hand, a recent clinical trial showed a beneficial effect of bosutinib, an oral dual Src/Bcr-Abl tyrosine kinase inhibitor, on TKV, but not on kidney function [24]. Src kinases may be both upstream and downstream of EGFR. Other investigators have argued that EGFR activation could be beneficial in the context of tissue repair following injury. This mechanism seems more likely, at least for ADPKD, since we found in the present study that decreased EGF was associated with more rapid kidney function decline instead of less rapid decline. Moreover, decreased renal expression and urinary excretion of EGF were found in human kidney diseases such as diabetic nephropathy, immunoglobulin A nephropathy, acute kidney injury and lupus nephritis, and in PKD, and were interpreted as a sign of insufficient repair [9, 25, 27]. Likewise, in patients with chronic kidney disease lower urinary EGF excretion was associated with a steeper slope of kidney function decline and worse renal outcome (defined as the incidence of end-stage kidney disease and/or a 40% reduction in eGFR), in both adults [28 and 29] as well as children [30, 31]. Finally, an experimental study has shown that EGF administration ameliorated cyst progression in PKD mice [32]. Taken together, the majority of these findings suggest that it is more likely that higher EGF may be beneficial in ADPKD, and thus support a protective role of EGFR activation in the kidney.

As we mentioned, urinary EGF is associated with eGFR decline in various other chronic kidney diseases besides ADPKD [22, 23]. These chronic kidney diseases have different underlying pathophysiology (with respect to the degree of inflammation, proliferation and fibrosis), which itself may have an effect on urinary EGF excretion. Keeping this in mind, we focused on the potential role of urinary EGF as a biomarker in ADPKD, and left a comparison of the predictive value of urinary EGF in ADPKD versus in other kidney diseases beyond the scope of the present study.

In our study, baseline urinary EGF was associated with future kidney function decline, but not with kidney growth. In patients with ADPKD, TKV is often used as proxy for kidney function. This practice is supported by the fact that during the natural course of ADPKD progression kidney size correlates well with present GFR as well as the rate of GFR decline over time [33–35]. However, for several interventions it has been shown that there is a lack of association between effects on kidney function and kidney volume [13, 36, 37]. Our observational data suggest that for the EGFR pathway there may be a similar dissociation, as also suggested by the aforementioned clinical study that showed for an EGFR pathway inhibitor disparate effects on kidney volume and growth.

Besides biomarkers that reflect glomerular function (eGFR), glomerular damage (albuminuria) and tubular damage (TKV in case of ADPKD), a clinical biomarker that reflects the amount of healthy kidney tissue would be welcome. Urinary EGF excretion might fill this gap. In this light, we studied the urinary excretion of EGF before and after kidney donation and noted that an approximately 50% reduction in kidney mass resulted in a similar reduction in urinary EGF. As expected, the effect of donating a kidney on GFR is far less pronounced due to compensatory hyperfiltration capacity of the remaining kidney. Prior studies have demonstrated that EGF is minimally detectable in plasma. EGF measured in urine is therefore produced by the kidney, and it has been shown that it originates especially from tubular cells [9, 29, 38]. Taking these findings into account, we interpret our findings that urinary EGF excretion might be a reflection of the amount of healthy tubular mass, and thus may be an indicator of tubular regenerative repair capacity as others have suggested [29].

Although EGF does not seem to be an important growth factor contributing to cyst growth in ADPKD, it is still not completely clarified whether the same holds for HB-EGF. Depending on the specific ligand that binds to the EGFR, different cellular responses can be activated [39, 40]. The exact underlying mechanism is unclear. One of the theories that has been put forward includes that each ligand induces different dimer pairs within the EGFR family which leads to a distinct downstream intracellular signal [40–44]. Considering these differences, the question remains of whether HB-EGF might be involved in ADPKD as a growth factor. A prior study indicated that HB-EGF leads to proliferation and is the most important ligand of the EGFR that might contribute to cyst growth due to its increased excretion associated with ADPKD disease severity [45]. The present data, however, contradict a significant detrimental effect of HB-EGF, given that urinary HB-EGF excretion was not associated with kidney function or with kidney function decline. Given this ambiguous preclinical observational evidence [45], future interventional research should resolve whether HB-EGF is a relevant factor in disease progression in ADPKD [45].

Our study has some limitations which we acknowledge. First, the number of subjects included in the longitudinal analyses is limited since we only included the 149 patients who were randomized to standard of care. We did so because lanreotide may influence disease progression. However, sensitivity analyses studying all 301 patients, independent of whether they received standard of care or lanreotide, showed similar results. Second, the follow-up period was limited to 2.5 years. Nevertheless, in this period there was considerable kidney function loss, with an annual rate of eGFR decline of –3.85 ± 2.05 mL/min/1.73 m^2^ per year, and we were able to show associations between urinary EGF excretion and this rate of eGFR loss. Longer follow-up is therefore expected to result only in stronger associations. Third, we did not collect data on plasma levels of EGF nor HB-EGF. Such data can be used to measure fractional urinary excretion of these markers. When values exceed 100%, it supports the notion that urinary EGF is of renal and not of systemic origin [9], which can shed more light on the role of EGF in the kidney. Finally, this study explored only associations between EGFR ligands and ADPKD progression. Establishing urinary EGF as a clinically applicable biomarker will require additional research, which is beyond the scope of the present study.

Strengths of this study include that data were obtained from a well-characterized ADPKD patient group with frequent follow-up visits, which allowed for a reliable calculation of eGFR slopes using mixed models. Furthermore, we measured urinary EGF excretions at baseline and after 12 weeks. The 12-week data provide clear evidence that EGF is rather stable over time in an individual, an important prerequisite when studying associations and developing compounds as biomarkers. Finally, we complemented our epidemiological findings with biopsy data studying renal expression of the EGFR, which were consistent with EGFR activation and thus support a role for the EGFR pathway in ADPKD.

In conclusion, our study shows that lower urinary EGF excretion is strongly associated with more severe disease at baseline, and with more rapid eGFR loss during follow-up. These data indicate that lower urinary EGF excretion may be a valuable biomarker to predict future kidney function decline in patients with ADPKD. We argue that lower urinary EGF excretion indicates that there is a less functional tubular mass and less regenerative capacity for repair.

## Supplementary Material

gfad050_Supplemental_File

## Data Availability

The data underlying this article will be shared on reasonable request to the corresponding author.

## Notes

Collaborating authors of the DIPAK consortium are listed in the Acknowledgments.
