# Supplementary material for: The association of urinary epidermal growth factors with ADPKD disease severity and progression
Source: Nephrol Dial Transplant. 2023 Mar 13;38(10):2266–75. doi: 10.1093/ndt/gfad050 (PMC10539218; doi:10.1093/ndt/gfad050)
Supplement: gfad050_Supplemental_File [file gfad050_supplemental_file.doc]

**Supplementary material**

**Table of content**

Page 3 **Supplementary Table 1.** Univariable associations of the 24-hour urinary excretion of epidermal growth factor (EGF) and heparin-binding EGF excretion (HB-EGF) with baseline characteristics of 301 ADPKD patients.

Page 4 **Supplementary Table 2.** Association of urinary epidermal growth factor (EGF) excretion and baseline total kidney volume in 301 ADPKD patients.

Page 5-6 **Supplementary Table 3A en B.** Association of urinary heparin-binding EGF excretion (uHB-EGF) with disease severity at baseline in 301 ADPKD patients.

Page 7 **Supplementary Table 4.** Baseline characteristics of 149 ADPKD patients receiving standard of care.

Page 8 **Supplementary Table 5.** Association of baseline urinary epidermal growth factor excretion (uEGF) with rate of kidney function decline in 152 ADPKD patients treated with lanreotide.

Page 9 **Supplementary Table 6.** Association of baseline urinary epidermal growth factor excretion corrected for creatinine excretion (uEGFcreat) with rate of kidney function decline during follow-up in 149 ADPKD patients receiving standard of care.

Page 10 **Supplementary Table 7.** Association of baseline urinary epidermal growth factor excretion (uEGF) with percentage of total kidney volume growth per year during follow-up in 149 ADPKD patients receiving standard of care.

Page 11 **Supplementary Table 8.** Association of baseline urinary heparin-binding EGF-like growth factor excretion (uHB-EGF) with rate of kidney function decline during follow-up in 149 ADPKD patients receiving standard of care .

Page 12 **Supplementary Table 9.** Association of baseline urinary heparin-binding EGF-like growth factor excretion (uHB-EGF) with total kidney volume growth expressed as percentage per year during follow-up in 149 ADPKD patients receiving standard of care.

Page 13 **Supplementary Figure 1.** Scatterplot of urinary epidermal growth factor excretion (uEGF) before and after kidney donation (n=72).

| **Supplementary Table 1.** Univariable associations of the 24-hour urinary excretion of epidermal growth factor (EGF) and heparin-binding EGF excretion (HB-EGF) with baseline characteristics of 301 ADPKD patients. | | | | | |
| --- | --- | --- | --- | --- | --- |
|  | **uEGF** (µg/24h) | |  | **uHB-EGF** (ng/24h) | |
|  | **R** | **p-value** |  | **R** | **p-value** |
| Age (years) | **-0.30** | **<0.001** |  | -0.05 | 0.44 |
| Sex, female | -0.02 | 0.72 |  | **-0.19** | **0.001** |
| BMI (kg/m^2^) | 0.10 | 0.10 |  | -0.05 | 0.36 |
| SBP (mmHg) | -0.01 | 0.84 |  | 0.06 | 0.30 |
| DBP (mmHg) | -0.02 | 0.68 |  | 0.09 | 0.14 |
| Use of antihypertensive drugs (% yes) | **-0.12** | **0.03** |  | **-0.11** | **0.05** |
| *PKD* mutation |  |  |  |  |  |
| PKD1 truncating | 0.03 | 0.65 |  | 0.02 | 0.83 |
| PKD1 non-truncating | -0.01 | 0.87 |  | -0.03 | 0.67 |
| htTKV (ml/m) | -0.03 | 0.60 |  | 0.11 | 0.06 |
| Mayo htTKV class |  |  |  |  |  |
| 1B + 1C | 0.05 | 0.71 |  | 0.20 | 0.14 |
| 1D + 1E | 0.07 | 0.59 |  | 0.23 | 0.10 |
| eGFR (mL/min per 1.73m^2^) | **0.56** | **<0.001** |  | **-0.16** | **0.006** |
| 24-hour urine volume (L) | -0.08 | 0.15 |  | 0.10 | 0.09 |
| Urinary EGF excretion (µg/24h) |  |  |  | 0.04 | 0.46 |
| Urinary HB-EGF excretion (ng/24h) | 0.04 | 0.46 |  |  |  |
| Assessed with use of Pearson’s correlation statistic. Data was logarithmically transformed if appropriate. Reference groups are PKD2 and others (non-PKD1 mutations) combined, and Mayo htTKV class 2 and 1A combined. Abbreviations: ADPKD, autosomal dominant polycystic kidney disease; BMI, body mass index; SBP, systolic blood pressure; DBP, diastolic blood pressure; htTKV, height adjusted total kidney volume; eGFR, estimated glomerular filtration rate using CKD-EPI equation; uHB-EGF, urinary heparin-binding EGF-like growth factor; uEGF, urinary epidermal growth factor. | | | | | |

| **Supplementary Table 2.** Association of urinary epidermal growth factor excretion (uEGF) with total kidney volume in 301 ADPKD patients. | | | | | | | | | | | | |
| --- | --- | --- | --- | --- | --- | --- | --- | --- | --- | --- | --- | --- |
|  | **Model 1** | | | **Model 2** | | | **Model 3** | | | **Model 4** | | |
|  | **St. β** | **p-value** | **R^2^** | **St. β** | **p-value** | **R^2^** | **St. β** | **p-value** | **R^2^** | **St. β** | **p-value** | **R^2^** |
| **Ln htTKV (mL/m)** |  |  | 0.10 |  |  | 0.14 |  |  | 0.16 |  |  | 0.28 |
| Ln uEGF (µg/24h) | -0.09 | 0.14 |  | 0.04 | 0.59 |  | 0.03 | 0.69 |  | -0.03 | 0.70 |  |
| Age (year) | -0.17 | 0.004 |  | -0.19 | 0.001 |  | -0.20 | 0.002 |  | -0.05 | 0.46 |  |
| Female Sex | 0.07 | <0.001 |  | -0.24 | <0.001 |  | -0.23 | <0.001 |  | -0.20 | 0.001 |  |
| eGFR (mL/min per 1.73m^2^) |  |  |  | -0.23 | 0.001 |  | -0.21 | 0.002 |  | -0.10 | 0.13 |  |
| *PKD* mutation |  |  |  |  |  |  |  |  |  |  |  |  |
| *PKD*1 truncating |  |  |  |  |  |  | -0.08 | 0.28 |  | -0.07 | 0.29 |  |
| *PKD*1 non-truncating |  |  |  |  |  |  | -0.18 | 0.01 |  | -0.15 | 0.02 |  |
| Urinary damage markers |  |  |  |  |  |  |  |  |  |  |  |  |
| β2MG (mcg/24h) |  |  |  |  |  |  |  |  |  | 0.03 | 0.61 |  |
| HFABP (µg/24h)* |  |  |  |  |  |  |  |  |  | -0.02 | 0.69 |  |
| MCP-1 (ng/24h) |  |  |  |  |  |  |  |  |  | 0.39 | <0.001 |  |
| Linear regression analysis with dependent variables indicated in bold. Data was logarithmically transformed if appropriate. Reference group for *PKD* mutation is *PKD*2 and others (non-*PKD* mutations) combined. Abbreviations: eGFR, estimated glomerular filtration rate; uEGF, urinary excretion of epidermal growth factor; htTKV, height adjusted total kidney volume; β2MG, β2-microglobulin HFABP, Heart-type Fatty Acid Binding Protein; MCP-1, monocyte chemotactic protein 1. | | | | | | | | | | | | |

| **Supplementary Table 3A.** Association of urinary heparin-binding EGF excretion (uHB-EGF) with disease severity at baseline in 301 ADPKD patients. | | | | | | | | | | | | |
| --- | --- | --- | --- | --- | --- | --- | --- | --- | --- | --- | --- | --- |
|  | **Model 1** | | | **Model 2** | | | **Model 3** | | | **Model 4** | | |
|  | **St. β** | **p-value** | **R^2^** | **St. β** | **p-value** | **R^2^** | **St. β** | **p-value** | **R^2^** | **St. β** | **p-val** | **R^2^** |
| **eGFR (mL/min/1.73m^2^)** |  |  | 0.08 |  |  | 0.12 |  |  | 0.12 |  |  | 0.23 |
| uHB-EGF (ng/24h) | -0.16 | 0.007 |  | -0.14 | 0.01 |  | -0.14 | 0.02 |  | -0.001 | 0.99 |  |
| Age (year) | -0.23 | <0.001 |  | -0.26 | <0.001 |  | -0.28 | <0.001 |  | -0.31 | <0.001 |  |
| Female Sex | 0.08 | 0.14 |  | 0.03 | 0.59 |  | 0.03 | 0.60 |  | 0.10 | 0.14 |  |
| Ln htTKV (mL/m) |  |  |  | -0.21 | 0.001 |  | -0.21 | 0.001 |  | -0.12 | 0.07 |  |
| *PKD* mutation |  |  |  |  |  |  |  |  |  |  |  |  |
| *PKD*1 truncating |  |  |  |  |  |  | -0.11 | 0.16 |  | -0.10 | 0.19 |  |
| *PKD*1 non-truncating |  |  |  |  |  |  | -0.02 | 0.80 |  | -0.006 | 0.93 |  |
| Urinary damage markers |  |  |  |  |  |  |  |  |  |  |  |  |
| Albumin (mg/24h) |  |  |  |  |  |  |  |  |  | 0.07 | 0.41 |  |
| IgG (mg/24h)* |  |  |  |  |  |  |  |  |  | -0.08 | 0.26 |  |
| β2MG (mcg/24h) |  |  |  |  |  |  |  |  |  | -0.01 | 0.85 |  |
| HFABP (µg/24h)* |  |  |  |  |  |  |  |  |  | -0.26 | <0.001 |  |
| NGAL (µg/24h) |  |  |  |  |  |  |  |  |  | -0.19 | 0.02 |  |
| MCP-1 (ng/24h) |  |  |  |  |  |  |  |  |  | -0.05 | 0.51 |  |
| Linear regression analysis with dependent variables indicated in bold. Data was logarithmically transformed if appropriate. Reference group for *PKD* mutation is *PKD*2 and others (non-*PKD* mutations) combined. Abbreviations: eGFR, estimated glomerular filtration rate; uHB-EGF, urinary excretion of heparin-binding EGF-like growth factor; uEGF, urinary excretion of epidermal growth factor; htTKV, height adjusted total kidney volume; β2MG, β2-microglobulin HFABP, Heart-type Fatty Acid Binding Protein; MCP-1, monocyte chemotactic protein 1. | | | | | | | | | | | | |

| **Supplementary Table 3B.** Association of urinary heparin-binding EGF excretion (uHB-EGF) with disease severity at baseline in 301 ADPKD patients. | | | | | | | | | | | | |
| --- | --- | --- | --- | --- | --- | --- | --- | --- | --- | --- | --- | --- |
|  | **Model 1** | | | **Model 2** | | | **Model 3** | | | **Model 4** | | |
|  | **St. β** | **p-value** | **R^2^** | **St. β** | **p-value** | **R^2^** | **St. β** | **p-value** | **R^2^** | **St. β** | **p-val** | **R^2^** |
| **Ln htTKV (mL/m)** |  |  | 0.10 |  |  | 0.14 |  |  | 0.16 |  |  | 0.29 |
| uHB-EGF (ng/24h) | 0.06 | 0.32 |  | 0.03 | 0.58 |  | 0.03 | 0.66 |  | -0.06 | 0.33 |  |
| Age (year) | -0.14 | 0.01 |  | -0.19 | 0.001 |  | -0.21 | 0.001 |  | -0.03 | 0.64 |  |
| Female Sex | -0.25 | <0.001 |  | -0.24 | <0.001 |  | -0.22 | <0.001 |  | -0.21 | 0.001 |  |
| eGFR (mL/min per 1.73m^2^) |  |  |  | -0.20 | 0.001 |  | -0.20 | 0.001 |  | -0.11 | 0.07 |  |
| *PKD* mutation |  |  |  |  |  |  |  |  |  |  |  |  |
| *PKD*1 truncating |  |  |  |  |  |  | -0.08 | 0.26 |  | -0.06 | 0.40 |  |
| *PKD*1 non-truncating |  |  |  |  |  |  | -0.18 | 0.01 |  | -0.15 | 0.03 |  |
| Urinary damage markers |  |  |  |  |  |  |  |  |  |  |  |  |
| Albumin (mg/24h) |  |  |  |  |  |  |  |  |  | 0.06 | 0.46 |  |
| IgG (mg/24h)* |  |  |  |  |  |  |  |  |  | 0.04 | 0.61 |  |
| β2MG (mcg/24h) |  |  |  |  |  |  |  |  |  | 0.02 | 0.84 |  |
| HFABP (µg/24h)* |  |  |  |  |  |  |  |  |  | -0.04 | 0.57 |  |
| NGAL (µg/24h) |  |  |  |  |  |  |  |  |  | 0.008 | 0.92 |  |
| MCP-1 (ng/24h) |  |  |  |  |  |  |  |  |  | 0.37 | <0.001 |  |
| Linear regression analysis dependent variables are indicated in bold. Data was logarithmically transformed if appropriate. Reference group for *PKD* mutation is *PKD*2 and others (non-*PKD* mutations) combined. Abbreviations: eGFR, estimated glomerular filtration rate; uHB-EGF, urinary excretion of heparin-binding EGF-like growth factor; uEGF, urinary excretion of epidermal growth factor; htTKV, height adjusted total kidney volume; β2MG, β2-microglobulin HFABP, Heart-type Fatty Acid Binding Protein; MCP-1, monocyte chemotactic protein 1. | | | | | | | | | | | | |

| **Supplementary Table 4.** Baseline characteristics of 149 ADPKD patients receiving standard of care. | |
| --- | --- |
| Age (years) | 49 ± 7.3 |
| Sex (% male) | 46 |
| BMI (kg/m^2^) | 26 [24 – 29] |
| SBP (mmHg) | 133 ± 14.0 |
| DBP (mmHg) | 82 ± 9.9 |
| Use of antihypertensive drugs (% yes) | 91 |
| PKD genotype (n (%)) |  |
| PKD1 truncating | 51 |
| PKD1 non-truncating | 26 |
| PKD2 | 17 |
| Others | 6 |
| htTKV (ml/m) | 1017 [713 – 1540] |
| Mayo htTKV class (n (%)) |  |
| 1A | 2 |
| 1B | 15 |
| 1C | 37 |
| 1D | 27 |
| 1E | 16 |
| 2 | 3 |
| eGFR (mL/min per 1.73m^2^) | 52 ± 11.6 |
| 24-hour urine volume (L) | 2.4 ± 0.84 |
| Urinary EGF excretion (µg/24h) | 18.8 [11.6 – 26.6] |
| Urinary EGF/creatinine ratio (µg/mmol) | 1.42 [0.91 – 2.18] |
| Urinary HB-EGF excretion (ng/24h) | 186 ± 104 |
| Urinary HB-EGF/creatinine ratio (ng/mmol) | 15.0 ± 9.4 |
| Variables are presented as mean ± SD when normally distributed and as median (interquartile range) when not normally distributed. Abbreviations: ADPKD, autosomal dominant polycystic kidney disease; BMI, body mass index; SBP, systolic blood pressure; DBP, diastolic blood pressure; htTKV, height adjusted total kidney volume; eGFR, estimated glomerular filtration rate using CKD-EPI equation; uHB-EGF, urinary heparin-binding EGF-like growth factor; uEGF, urinary epidermal growth factor. | |

| **Supplementary Table 5.** Association of baseline urinary epidermal growth factor excretion (uEGF) with rate of kidney function decline in 152 ADPKD patients treated with lanreotide. | | | | | | | | | | | | |
| --- | --- | --- | --- | --- | --- | --- | --- | --- | --- | --- | --- | --- |
|  | **Crude** | | **Model 1** | | **Model 2** | | **Model 3** | | **Model 4** | | **Model 5** | |
|  | **β** | **p-value** | **β** | **p-value** | **β** | **p-value** | **β** | **p-value** | **β** | **p-value** | **β** | **p-value** |
| **Change in eGFR (mL/min/1.73m^2^/year)** |  |  |  |  |  |  |  |  |  |  |  |  |
| Ln uEGF (µg/24h) | 0.63 | 0.04 | 0.77 | 0.012 | 0.88 | 0.02 | 0.94 | 0.01 | 0.92 | 0.015 | 1.20 | 0.001 |
| Age (per 10 years) |  |  | 0.81 | 0.002 | 0.81 | 0.002 | 0.76 | 0.02 | 0.71 | 0.03 | 0.22 | 0.49 |
| Female Sex |  |  | 0.13 | 0.71 | 0.14 | 0.70 | 0.13 | 0.74 | 0.25 | 0.52 | 0.01 | 0.98 |
| eGFR (per 10 mL/min/1.73m^2^) |  |  |  |  | -0.09 | 0.65 | -0.04 | 0.83 | -0.06 | 0.78 | -0.50 | 0.015 |
| Mayo htTKV class |  |  |  |  |  |  |  |  |  |  |  |  |
| 1B + 1C |  |  |  |  |  |  | -0.95 | 0.29 | -0.75 | 0.41 | -0.73 | 0.38 |
| 1D + 1E |  |  |  |  |  |  | -1.01 | 0.30 | -0.78 | 0.43 | -0.51 | 0.57 |
| *PKD* mutation |  |  |  |  |  |  |  |  |  |  |  |  |
| *PKD*1 truncating |  |  |  |  |  |  |  |  | -0.40 | 0.41 | -0.23 | 0.61 |
| *PKD*1 non-truncating |  |  |  |  |  |  |  |  | -0.56 | 0.30 | -0.47 | 0.34 |
| Urinary damage markers |  |  |  |  |  |  |  |  |  |  |  |  |
| β2MG (mcg/24h) |  |  |  |  |  |  |  |  |  |  | 0.07 | 0.73 |
| HFABP (µg/24h) |  |  |  |  |  |  |  |  |  |  | -1.05 | 0.01 |
| MCP-1 (ng/24h) |  |  |  |  |  |  |  |  |  |  | -1.20 | <0.001 |
| Associations tested with mixed model analysis. uEGF was ln transformed to attain normal distribution. Reference groups are PKD2 and other (non-PKD1 mutations) combined, and Mayo htTKV class 2 and 1A combined. Abbreviations: eGFR, estimated glomerular filtration rate; uEGF, urinary EGF excretion; htTKV, height adjusted total kidney volume; β2MG, β2-microglobulin; HFABP, Heart-type Fatty Acid Binding Protein; MCP-1, monocyte chemotactic protein 1. | | | | | | | | | | | | |

| **Supplementary Table 6.** Association of baseline urinary epidermal growth factor excretion corrected for creatinine excretion (uEGFcreat) with rate of kidney function decline during follow-up in 149 ADPKD patients receiving standard of care. | | | | | | | | | | | | |
| --- | --- | --- | --- | --- | --- | --- | --- | --- | --- | --- | --- | --- |
|  | **Crude** | | **Model 1** | | **Model 2** | | **Model 3** | | **Model 4** | | **Model 5** | |
|  | **β** | **p-value** | **β** | **p-value** | **β** | **p-value** | **β** | **p-value** | **β** | **p-value** | **β** | **p-value** |
| **Change in eGFR (mL/min/1.73m^2^/year)** |  |  |  |  |  |  |  |  |  |  |  |  |
| uEGFcreat (ng/mmol) | 1.31 | <0.001 | 1.66 | <0.001 | 1.75 | <0.001 | 1.73 | <0.001 | 1.71 | <0.001 | 1.51 | <0.001 |
| Age (per 10 years) |  |  | 1.19 | <0.001 | 1.13 | <0.001 | 0.79 | 0.01 | 0.46 | 0.17 | 0.26 | 0.44 |
| Female Sex |  |  | -0.39 | 0.32 | -0.46 | 0.25 | -0.57 | 0.15 | -0.41 | 0.30 | -0.52 | 0.21 |
| eGFR (per 10 mL/min/1.73m^2^) |  |  |  |  | -0.09 | 0.63 | -0.17 | 0.38 | -0.11 | 0.58 | -0.23 | 0.23 |
| Mayo htTKV class |  |  |  |  |  |  |  |  |  |  |  |  |
| 1B + 1C |  |  |  |  |  |  | -1.02 | 0.22 | -1.08 | 0.19 | -0.74 | 0.37 |
| 1D + 1E |  |  |  |  |  |  | -1.97 | 0.03 | -2.17 | 0.01 | -1.73 | 0.05 |
| *PKD* mutation |  |  |  |  |  |  |  |  |  |  |  |  |
| *PKD*1 truncating |  |  |  |  |  |  |  |  | -1.18 | 0.02 | -1.37 | 0.008 |
| *PKD*1 non-truncating |  |  |  |  |  |  |  |  | -1.23 | 0.03 | -1.42 | 0.01 |
| Urinary damage markers |  |  |  |  |  |  |  |  |  |  |  |  |
| β2MG (mcg/24h) |  |  |  |  |  |  |  |  |  |  | -0.10 | 0.62 |
| HFABP (µg/24h) |  |  |  |  |  |  |  |  |  |  | -0.68 | 0.04 |
| MCP-1 (ng/24h) |  |  |  |  |  |  |  |  |  |  | -0.34 | 0.19 |
| Associations tested with mixed model analysis. uEGFc was ln transformed to attain normal distribution. Reference groups are PKD2 and other (non-PKD1 mutations) combined, and Mayo htTKV class 2 and 1A combined. Abbreviations: eGFR, estimated glomerular filtration rate, uEGFreat, urinary epidermal growth factor excretion corrected for urinary creatinine excretion, htTKV, height adjusted total kidney volume; β2MG, β2-microglobulin HFABP, Heart-type Fatty Acid Binding Protein; MCP-1, monocyte chemotactic protein 1. | | | | | | | | | | | | |
| **Supplementary Table 7.** Association of baseline urinary epidermal growth factor excretion (uEGF) with percentage of total kidney volume growth per year during follow-up in 149 ADPKD patients receiving standard of care. | | | | | | | | | | | | |
|  | **Crude** | | **Model 1** | | **Model 2** | | **Model 3** | | **Model 4** | | **Model 5** | |
|  | **St. β** | **p-value** | **St. β** | **p-value** | **St. β** | **p-value** | **St. β** | **p-value** | **St. β** | **p-value** | **St. β** | **p-value** |
| **Change in TKV (%/year)** |  |  |  |  |  |  |  |  |  |  |  |  |
| Ln uEGF (µg/24h) | -0.009 | 0.92 | -0.06 | 0.51 | -0.01 | 0.90 | -0.01 | 0.93 | -0.02 | 0.89 | -0.03 | 0.76 |
| Age (per 10 years) |  |  | -0.09 | 0.35 | -0.10 | 0.29 | -0.08 | 0.49 | -0.14 | 0.25 | -0.12 | 0.33 |
| Female Sex |  |  | -0.31 | 0.001 | -0.28 | 0.002 | -0.27 | 0.003 | -0.27 | 0.004 | -0.29 | 0.005 |
| eGFR (per 10 mL/min/1.73m^2^) |  |  |  |  | -0.13 | 0.21 | -0.12 | 0.24 | -0.17 | 0.11 | -0.16 | 0.16 |
| Mayo htTKV class |  |  |  |  |  |  |  |  |  |  |  |  |
| 1B + 1C |  |  |  |  |  |  | 0.06 | 0.76 | 0.09 | 0.61 | 0.07 | 0.71 |
| 1D + 1E |  |  |  |  |  |  | 0.11 | 0.58 | 0.16 | 0.42 | 0.11 | 0.61 |
| *PKD* mutation |  |  |  |  |  |  |  |  |  |  |  |  |
| *PKD*1 truncating |  |  |  |  |  |  |  |  | -0.11 | 0.39 | -0.10 | 0.43 |
| *PKD*1 non-truncating |  |  |  |  |  |  |  |  | 0.03 | 0.82 | 0.03 | 0.82 |
| Urinary damage markers |  |  |  |  |  |  |  |  |  |  |  |  |
| β2MG (mcg/24h) |  |  |  |  |  |  |  |  |  |  | 0.06 | 0.58 |
| HFABP (µg/24h) |  |  |  |  |  |  |  |  |  |  | -0.04 | 0.72 |
| MCP-1 (ng/24h) |  |  |  |  |  |  |  |  |  |  | 0.07 | 0.54 |
| Associations tested with linear regression analysis with depended variable indicated in bold. Percentage change per year in TKV was calculated as ((TKV last study visit / TKV baseline visit)^1 / total study time^ ) – 1 *100. uEGF was ln transformed to attain normal distribution. Reference groups are PKD2 and other (non-PKD1 mutations) combined, and Mayo htTKV class 2 and 1A combined. Abbreviations: eGFR, estimated glomerular filtration rate; uEGF, urinary EGF excretion; htTKV, height adjusted total kidney volume; β2MG, β2-microglobulin; HFABP, Heart-type Fatty Acid Binding Protein; MCP-1, monocyte chemotactic protein 1. | | | | | | | | | | | | |
| **Supplementary Table 8.** Association of baseline urinary heparin-binding EGF-like growth factor excretion (uHB-EGF) with rate of kidney function decline during follow-up in 149 ADPKD patients receiving standard of care. | | | | | | | | | | | | |
|  | **Crude** | | **Model 1** | | **Model 2** | | **Model 3** | | **Model 4** | | **Model 5** | |
|  | **β** | **p-value** | **β** | **p-value** | **β** | **p-value** | **β** | **p-value** | **β** | **p-value** | **β** | **p-value** |
| **Change in eGFR (mL/min/1.73m^2^/year)** |  |  |  |  |  |  |  |  |  |  |  |  |
| uHB-EGF (ng/24h) | -0.002 | 0.28 | -0.002 | 0.33 | -0.002 | 0.45 | -0.001 | 0.51 | -0.002 | 0.35 | 0.001 | 0.67 |
| Age (per 10 years) |  |  | 0.85 | 0.003 | 0.97 | 0.001 | 0.60 | 0.07 | 0.30 | 0.40 | 0.13 | 0.72 |
| Female Sex |  |  | 0.11 | 0.81 | -0.05 | 0.90 | -0.20 | 0.64 | -0.06 | 0.89 | -0.14 | 0.77 |
| eGFR (per 10 mL/min/1.73m^2^) |  |  |  |  | 0.32 | 0.09 | 0.24 | 0.20 | 0.32 | 0.09 | 0.07 | 0.70 |
| Mayo htTKV class |  |  |  |  |  |  |  |  |  |  |  |  |
| 1B + 1C |  |  |  |  |  |  | -1.41 | 0.11 | -1.55 | 0.08 | -1.17 | 0.18 |
| 1D + 1E |  |  |  |  |  |  | -2.37 | 0.01 | -0.267 | 0.005 | -2.16 | 0.02 |
| *PKD* mutation |  |  |  |  |  |  |  |  |  |  |  |  |
| *PKD*1 truncating |  |  |  |  |  |  |  |  | -1.10 | 0.05 | -1.36 | 0.02 |
| *PKD*1 non-truncating |  |  |  |  |  |  |  |  | -1.47 | 0.02 | -1.65 | 0.006 |
| Urinary damage markers |  |  |  |  |  |  |  |  |  |  |  |  |
| Albumin (mg/24h) |  |  |  |  |  |  |  |  |  |  | 0.49 | 0.50 |
| IgG (mg/24h) |  |  |  |  |  |  |  |  |  |  | -0.40 | 0.17 |
| β2MG (mcg/24h) |  |  |  |  |  |  |  |  |  |  | -0.08 | 0.77 |
| HFABP (µg/24h) |  |  |  |  |  |  |  |  |  |  | -1.04 | 0.01 |
| NGAL (µg/24h) |  |  |  |  |  |  |  |  |  |  | -0.17 | 0.72 |
| MCP-1 (ng/24h) |  |  |  |  |  |  |  |  |  |  | -0.27 | 0.36 |
| Associations tested with mixed model analysis. Reference groups are PKD2 and other (non-PKD1 mutations) combined, and Mayo htTKV class 2 and 1A combined. Abbreviations; eGFR, estimated glomerular filtration rate, uHB-EGF, urinary heparin-binding EGF-like growth factor excretion, htTKV, height adjusted total kidney volume; β2MG, β2-microglobulin HFABP, Heart-type Fatty Acid Binding Protein; MCP-1, monocyte chemotactic protein 1. | | | | | | | | | | | | |

| **Supplementary Table 9.** Association of baseline urinary heparin-binding EGF-like growth factor excretion (uHB-EGF) with total kidney volume growth expressed as percentage per year during follow-up in 149 ADPKD patients receiving standard of care. | | | | | | | | | | | | |
| --- | --- | --- | --- | --- | --- | --- | --- | --- | --- | --- | --- | --- |
|  | **Crude** | | **Model 1** | | **Model 2** | | **Model 3** | | **Model 4** | | **Model 5** | |
|  | **St. β** | **p-value** | **St. β** | **p-value** | **St. β** | **p-value** | **St. β** | **p-value** | **St. β** | **p-value** | **St. β** | **p-value** |
| **Change in TKV (%/year)** |  |  |  |  |  |  |  |  |  |  |  |  |
| uHB-EGF (ng/24h) | 0.04 | 0.70 | -0.06 | 0.52 | -0.08 | 0.36 | -0.08 | 0.36 | -0.06 | 0.51 | -0.11 | 0.30 |
| Age (per 10 years) |  |  | -0.06 | 0.50 | -0.10 | 0.30 | -0.07 | 0.50 | -0.13 | 0.25 | -0.14 | 0.27 |
| Female Sex |  |  | -0.32 | <0.001 | -0.30 | 0.001 | -0.29 | 0.002 | -0.29 | 0.003 | -0.23 | 0.06 |
| eGFR (per 10 mL/min/1.73m^2^) |  |  |  |  | -0.14 | 0.13 | -0.13 | 0.17 | -0.18 | 0.07 | -0.19 | 0.07 |
| Mayo htTKV class |  |  |  |  |  |  |  |  |  |  |  |  |
| 1B + 1C |  |  |  |  |  |  | 0.06 | 0.74 | 0.10 | 0.60 | 0.06 | 0.76 |
| 1D + 1E |  |  |  |  |  |  | 0.11 | 0.56 | 0.16 | 0.40 | 0.08 | 0.71 |
| *PKD* mutation |  |  |  |  |  |  |  |  |  |  |  |  |
| *PKD*1 truncating |  |  |  |  |  |  |  |  | -0.11 | 0.39 | -0.12 | 0.38 |
| *PKD*1 non-truncating |  |  |  |  |  |  |  |  | 0.03 | 0.84 | 0.03 | 0.79 |
| Urinary damage markers |  |  |  |  |  |  |  |  |  |  |  |  |
| Albumin (mg/24h) |  |  |  |  |  |  |  |  |  |  | -0.16 | 0.28 |
| IgG (mg/24h) |  |  |  |  |  |  |  |  |  |  | 0.09 | 0.43 |
| β2MG (mcg/24h) |  |  |  |  |  |  |  |  |  |  | 0.16 | 0.21 |
| HFABP (µg/24h) |  |  |  |  |  |  |  |  |  |  | 0.10 | 0.42 |
| NGAL (µg/24h) |  |  |  |  |  |  |  |  |  |  | -0.17 | 0.29 |
| MCP-1 (ng/24h) |  |  |  |  |  |  |  |  |  |  | 0.14 | 0.24 |
| Associations tested with linear regression analysis. Percentage change per year in TKV was calculated as ((TKV last study visit / TKV baseline visit)^1 / total study time^ ) – 1 *100. Reference groups are PKD2 and other (non-PKD1 mutations) combined, and Mayo htTKV class 2 and 1A combined. Abbreviations; eGFR, estimated glomerular filtration rate, uHB-EGF, urinary heparin-binding EGF-like growth factor excretion, htTKV, height adjusted total kidney volume; β2MG, β2-microglobulin HFABP, Heart-type Fatty Acid Binding Protein; MCP-1, monocyte chemotactic protein 1. | | | | | | | | | | | | |

**Supplementary Figure 1.** Scatterplot of urinary epidermal growth factor excretion (uEGF) before and 5.5 [4.0 – 7.0] months after kidney donation (n=72). The solid line is drawn with use of Deming regression and the dotted line represents the line of identity. The correlation coefficient was determined with Pearson’s correlation statistics. On average, kidney donation resulted in a change of uEGF of -46.4 [-63.3 to -17.6] %.
